# Rheological and Functional Properties of Mechanically Recycled Post-Consumer Rigid Polyethylene Packaging Waste

**DOI:** 10.3390/ma17081855

**Published:** 2024-04-17

**Authors:** Ezgi Ceren Boz Noyan, Franziska Rehle, Antal Boldizar

**Affiliations:** 1 Department of Industrial and Materials Science, Chalmers University of Technology, Rännvägen 2A, SE-41296 Gothenburg, Sweden; ezgi.boznoyan@chalmers.se (E.C.B.N.); franziska.rehle@polymtl.ca (F.R.); 2 The International Reference Center for Life Cycle Assessment and Sustainable Transition (CIRAIG), Chemical Engineering Department, Polytechnique Montreal, 3333 Queen Mary Rd Suite 310, Montreal, QC H3V 1A2, Canada

**Keywords:** rigid plastic packaging waste, polyethylene, plastic recycling, rheological properties, washing, melt strength, mechanical properties

## Abstract

The properties of recycled post-consumer rigid polyethylene packaging waste were studied, using sorted waste washed in the laboratory with water alone and with added detergent, and compared with large-scale high-intensity washed flakes. The washed flakes were compounded using three different temperature profiles in a twin-screw extruder and then injection molded. A higher compounding temperature reduced the thermo-oxidative stability, the average molecular mass, and the viscosity of the samples. Rheological measurements suggested that changes in chain branching occurred at different compounding temperatures. The strength and the elongation at break were also influenced by the compounding temperature in both the molten and solid states. Detergent washing maintained the thermo-oxidative stability in contrast to washing with water. The large-scale washed samples had a relatively high thermo-oxidative stability, a higher melt elasticity, and a lower elongation at break in both the molten and solid states than the laboratory-scale washed samples. The thermal properties, melt elasticity, Young’s modulus, yield stress, and yield strain of the samples were not, however, significantly affected by either the compounding temperature or the washing medium and intensity. The results indicated that recycled post-consumer rigid polyethylene packaging waste has properties that can support further applications in new products.

## 1. Introduction

For resource efficiency and to reach the European Union (EU) recycling targets for packaging plastics [1], mechanical recycling is the most relevant strategy if reuse is not possible. Mechanical recycling is economically and environmentally more efficient than other currently available recycling technologies, but to increase the amount of recycled plastics as well as the recyclate quality and to facilitate multiple-loop recycling of plastics used for fast-moving consumer goods (FMCGs), issues such as polymer degradation, inconsistency of product properties, and contamination must be addressed [2,3]. For many decades, packaging has been the largest application for plastics in Europe as well as globally, mainly in the use of polyethylene and polypropylene for short-lived packaging materials [4]. Thus, there is a great need to increase high-quality mechanical recycling of these materials.

Bales of automatically sorted post-consumer packaging plastics are known to contain a variety of contaminants after leaving the material recovery facilities [5,6,7]. These contaminants influence properties of the recyclate such as color, gloss, and odor, and they lead to quality variations, which are problematic for subsequent industrial applications [7,8]. For this reason, sorted post-consumer plastics are commonly washed before further processing [3,9], although washing residues can lead to degradation during the subsequent processing steps [10]. Santana and Gondim [10] reported that the oxidative degradation of post-consumer HDPE samples was related to the agent used for washing. They compared water-based washing with washing with caustic soda or a detergent, and they found that NaOH-washed HDPE samples deteriorated more than samples treated with detergent or a 1:1 NaOH–detergent mix.

Mechanical recycling also leads to more extensive deterioration of the polymeric material than that caused by manufacturing and use [11,12], due mainly to thermal or oxidative degradation or a combination of both [13,14]. Thermo-oxidative degradation occurs when a polymer is exposed to oxygen, either as crosslinking and branching or as chain scission [10,15]. Thermo-oxidation, the most relevant type of chemical degradation, occurs predominantly as a thermally activated radical formation, following a mechanism similar to polymerization, and, depending on the predominant reactions, can lead either to an increase in molecular weight due to recombination or to a decrease due to fragmentation [14].

It is known that during the reprocessing of HDPE, thermo-oxidative degradation involves chain scission, chain-branching, and crosslinking and that these reactions may occur simultaneously depending on the polymer synthesis method, the molecular structure of the polymer, the washing conditions, the compounding temperature, and the number of extrusion cycles [2,10,11,16,17,18,19,20,21,22,23,24,25]. Rheological characterization can be used both to relate the melt behavior to the processability and to investigate the degradation-related changes in the polymer structure. The viscosity curve from capillary rheometry can provide an indication of different degradation levels due to different processing conditions since more degraded material usually shows a lower viscosity [21]. The entrance pressure losses during the capillary flow can be associated with the melt elasticity, which is important for melt processing, e.g., extrusion, injection molding, film blowing, and blow molding, indicating aspects of processability such as swelling ratio, haze formation, etc. [26,27,28,29,30]. Rheotens measurements in connection with capillary rheometry are useful to indicate the melt strength and drawability of a polymer melt, which can be important for many polymer processes [31]. This type of rheological property is sensitive to the molecular structure, which leads to different melt characteristics depending on the branching type, topology, and amount and also on the polymer properties [32].

Dynamic shear measurements by rotational rheometry have been extensively used to establish relationships between rheological properties and polymer structure. van Gurp and Palmen [33] studied the time–temperature superposition of different polymeric blends and showed that the development of the phase angle versus the complex shear modulus, referred to as a van Gurp–Palmen plot (vGP plot), was characteristic of the chemical nature of the polymer [34]. Several studies have reported that linear polymers usually exhibit a monotonic decrease in a vGP plot, whereas a polymer with long-chain branching (LCB) exhibits a different shape with a bump or a plateau in the phase angle–modulus curve with a magnitude and breadth depending on the degree of LCB [34,35,36,37,38]. A plateau in the phase angle in a vGP plot has been attributed by Shahi et al. [37] to the existence of a gel structure or light cross-linking. Trinkle et al. [39], in a study of LCB polymers with different topologies, concluded that the position of the branches along the chain had a greater impact on the rheological behavior than the number of branches. The phase angle in a vGP plot was shifted to a lower value with an increasing level of LCB [20,35,36,38]. It was possible to determine the viscous to elastic transition using a vGP plot where a phase angle below 45° indicates a predominant elasticity [37]. Lower values of the phase angle indicate a more elastic behavior [35], which has also been attributed to the formation of a 3D interlocked structure [37] and to the formation of branching due to degradation [20]. A more elastic behavior is also reflected in a lower crossover frequency and a higher storage modulus at a low frequency level, which can be ascribed to a greater level of branching [20,36,37]. It has also been observed that the zero-shear viscosity and the complex viscosity at lower frequency levels tend to increase with increasing levels of LCB [20,36,38].

With this background, the objective of this work was to investigate the influence of the processing conditions on the rheological properties of the mechanically recycled rigid PE stream from post-consumer plastic packaging waste. The focus was on assessing degradation-related structural changes in recycled materials washed and extruded under different conditions. The mechanical properties of injection-molded samples of the recyclates are also commented on.

## 2. Materials and Methods

### 2.1. Materials and Chemicals

Sorted post-consumer rigid PE waste (PE-rigid) was received from two large-scale plants. One type, originating from source-separated household plastic packaging waste, was supplied from a sorting plant in Motala, Sweden (plant S) in the autumn of 2020, and the other type, originating from mixed municipal solid waste, was supplied from a sorting, washing, and compounding plant in Stavanger, Norway (plant N) in the spring of 2021. From plant N, ca. 60 kg of washed flakes were also received. For the laboratory-scale washing, a detergent (D) “Via professional liquid colour, perfume free” (Unilever Professional), containing 5–15% of anionic surfactants, less than 5% non-ionic surfactants and soap, and less than 1% enzymes, phenoxyethanol, and methylisothiazolinone, was used as washing agent.

### 2.2. Sampling of Waste Samples for Feedstock Characterization

To characterize the feedstock of the sorted PE-rigid stream from plant S, a sampling was performed over a five-week period on two or three days each week. Samples of 200 L were taken, resulting in eight bags of sorted plastic pieces, which were used both for feedstock characterization and for further processing in the laboratory. From a bale of ca. 800 kg of sorted PE-rigid from plant N, a 200 L sample was taken for feedstock characterization, and ca. 120 kg was taken separately from the same bale for processing in the laboratory. The eight samples of the sorted PE-rigid stream from plant S and one sample from plant N, each sample consisting of 100 pieces, were analyzed using a handheld near-infrared (NIR) analyzer of the microPHAZIR-Thermo type (Waltham, MA, USA).

### 2.3. Washing

**Figure 1:**
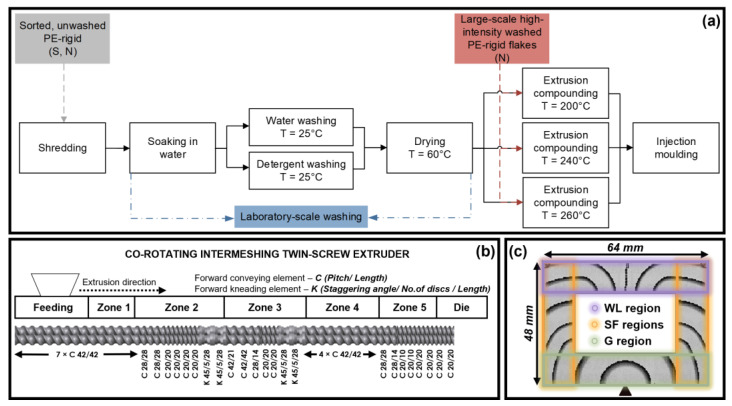
(**a**) Flow chart of the laboratory-scale processing, (**b**) the configuration of the co-rotating intermeshing twin-screw used for compounding, and (**c**) the filling pattern in the mold producing a specimen with a thickness of 2 mm.

The sorted plastics waste was first shredded using a Rapid Granulator 300-45KU (Bredaryd, Sweden) with a sieve size of 17 mm, and the shredded flakes were then soaked, washed, and dried prior to extrusion compounding. The laboratory-scale sample preparation is shown in Figure 1a, where Figure 1b illustrates the screw configuration used for the extrusion compounding, and Figure 1c shows the filling pattern of the injection-molded sample. The same procedure was adopted for both the unwashed PE materials from the plants S and N.

A 1 kg sample of the shredded plastic waste was soaked and agitated in a metal tub containing 60 L of tap water at room temperature. Two batches of the floating fraction were used in each washing cycle in a Vortex M6, SDL Atlas machine (Rockhill, SC, USA) using 72 L of water at 25 °C. In each washing cycle, the agitation, drainage, and rinsing lasted for ca. 45 min. Washings were performed with water alone or with 0.4 wt.% added detergent in 72 L of water. Three or four batches of the machine-washed flakes of each type were dried in a Moretto SX201 dryer (Massanzago, Italy) at 60 °C overnight to 4% RH recorded by the EL-USB-2-LCD Lascar Electronics hygrometer placed inside the dryer (Whiteparish, UK).

The washed flakes from plant N were used as received. The washing procedure involved shredding, magnetic screening, washing at room temperature, wet-grinding, friction hot washing at 70–80 °C, rinsing at room temperature, centrifuging, and finally drying, according to the supplier. The PE-films were separated from the PE-rigids via the wind sifter after drying. The whole process took approximately 30 min. The medium used in the friction hot washing contained NaOH, defoamer, detergent, and other additives. The results are henceforth called the large-scale high-intensity washed sample.

### 2.4. Extrusion Compounding and Injection Molding

The washed flakes were compounded using a Werner & Pfleiderer ZSK 30 M9/2 co-rotating intermeshing twin-screw extruder (TSE) (Stuttgart, Germany), with a screw length of 969 mm and a diameter of 30 mm. For the samples washed in the laboratory with water alone and the samples washed on a large scale with high intensity, three different compounding temperature profiles were used, 110-160-200-200-200, 110-160-200-240-240, and 110-160-210-260-260 °C from heating zones 1 to 5 shown in Figure 1b, with die temperatures of 210, 250, and 270 °C. For the samples washed with added detergent an intermediate temperature profile (110-160-200-240-240 °C in the zones and 250 °C at the die) was used. Here, 3–4 kg of each material were extruded at a rate of 2.1 ± 0.4 kg/h with a screw rotation rate of 80 rpm. The compounded strands were pelletized using a Dreher pelletizer type SG10Ni (Aachen, Germany). 

An Arburg Allrounder 221M-250-5 machine (Lossburg, Germany) was used to injection mold samples with the shape of a frame (Figure 1c), providing three sample regions with different structures. The gate (G) region had a mixed molecular orientation, the simple flow (SF) region had a predominantly unidirectional structure, and the weld line (WL) region had a structure dominated by the meeting of the two melt flow fronts. The injection molding was conducted with a temperature profile of 120-160-200-240 °C along the barrel and 240 °C at the nozzle, and the injection and holding pressures were 500 and 900 bar, respectively.

The notations of the specimens produced are given in Table 1, indicating their processing histories.

### 2.5. Characterization

#### 2.5.1. Thermal Properties

The thermal transitions and the oxidation induction temperature (*T_ox_*) of the compounded pellets and injection-molded samples were assessed by differential scanning calorimetry (DSC) using a Mettler-Toledo DSC 2 (Columbus, OH, USA). The samples were prepared according to [40] and [41]. The thermal transitions were measured in a nitrogen atmosphere with heating and cooling rates of 10 °C/min, and the *T_ox_* measurements were performed using air as a purging gas at a heating rate of 10 °C/min. Duplicates were made in all cases. At measuring, for the heat of fusion (Δ*H*), the baseline was taken from 65 to 144 °C, and the results were reported for the first heating cycles. The degree of crystallinity (*X_c_*) was assessed as *X_c_* = ((Δ*H*/(*w_PE-rigid_ ×* Δ*H*_0_)) × 100) (%), where *w_PE-rigid_* is the weight fraction of PE-rigid taken from the feedstock characterization for each type of material and the heat of fusion for 100% crystalline polyethylene Δ*H*_0_ taken as 293 J/g [8,42].

The ash content of the ground pellets was determined by thermogravimetric analysis (TGA) using a TGA/DSC 3+ Star system from Mettler Toledo (Columbus, OH, USA), where the 3.1 ± 0.1 mg samples were heated from 25 °C to 650 °C at a rate of 10 °C/min in air at a flow rate of 50 mL/min. Duplicate measurements were performed for each type of material, and the ash content values were determined at 600 °C.

#### 2.5.2. Molecular Structure

High-temperature gel permeation chromatography (HT-GPC) was performed using a Polymer Laboratories GPC220 instrument (Santa Clara, CA, USA) with PlOlexis and PlOlexis guard columns with lengths of 3 × 30 cm at 160 °C, an injection volume of 200 μL, and a flow rate of 0.8 mL/min. Samples were dissolved in 1,2,4 trichlorobenzene with 200 ppm butylated hydroxytoluene (BHT) as an antioxidant at a concentration of 3 mg/mL. The results given are the weight average molecular mass (*M_w_*) and the polydispersity (*PD*) based on two independent measurements except for the laboratory-scale sample from plant S that was washed with detergent for which only one measurement was performed.

#### 2.5.3. Rheological Properties

The melt-mass-flow rate (*MFR*) of the pellets was determined using a Ceast Modular Melt Flow instrument (Instron/Ceast, Pianezza, Italy) with a weight of 2.16 kg at 240 °C in accordance with the [43].

A high-pressure Rheograph 20 capillary rheometer (Göttfert, Buchen, Germany) was used to evaluate the melt viscosity of the materials at shear rates between 1 and 10^3^ s^−1^ at 240 °C. The Bagley correction [44] was applied using three dies having a diameter (D) of 2 mm and aspect ratios (L/D) of 5, 10, and 15. The Weissenberg–Rabinowitsch shear rate correction [44] was also applied. The graphs of corrected viscosity versus shear rate and of the entrance pressure losses based on the Bagley plots are given for the die with an L/D ratio of 10.

Dynamic-oscillatory shear flow measurements were performed using an Anton Paar MCR702 rheometer (Graz, Austria) with a 25 mm parallel plate geometry. Laboratory-washed samples from plant N were used for this characterization, and 25 mm discs were prepared using an Xplore micro injection molder IM12 (Sittard, The Netherlands) and conditioned for at least 48 h at 21 ± 1 °C and 55 ± 5% RH prior to the measurements. The measuring gap was set to 1 mm, and all the measurements were performed at 180 °C in a nitrogen atmosphere. Oscillatory strain sweep tests were first performed at a constant frequency of 1 Hz in the shear strain range 0.001–100% to determine the linear viscoelastic (LVE) region for each sample. Frequency sweep tests were then carried out at a constant shear strain of 1% on the LVE within a frequency range of 100–0.0016 Hz. The storage modulus (*G′*), the loss modulus (*G″*), the complex modulus (G*), the complex viscosity (η*), and the phase angle (*δ*) were recorded.

The same capillary rheometer equipped with a haul-off unit consisting of a strand wheel connected to a force transducer and a take-off wheel was used to determine the elongational properties of the melts. A capillary with a diameter of 2 mm and a length of 20 mm was used at a melt temperature of 240 °C in an ambient environment of 24 ± 1 °C and 27 ± 3% relative humidity, following [45] as a guideline. The drawing length was 220 mm. The initial velocity (*v*_0_) of the extruded strand at the exit of the capillary was kept constant at 13.5 mm/s, and the starting tangential velocity of the take-off wheel was 13.5 mm/s for all the samples. The speed (*v*_1_) of the take-off wheel increased with an acceleration rate of 0.24 mm/s^2^. The force required to extend the melt was measured together with the time and velocity of the take-off wheel until the extended strand broke. The strain (*ε*) was calculated as *ε* = (*v*_1_ *− v*_0_*)/v*_0_ [46]. The mean melt strength (force at break) and the mean strain at break were based on at least five independent measurements.

#### 2.5.4. Mechanical Properties

A Zwick/Roell Z2.5 tensile tester equipped (Ulm, Germany) with a 2 kN load cell was used to measure the tensile properties of the injection-molded samples. Test bars of type 5A in [47] were cut from the three different regions of the molded frames and conditioned according to [48]. The Young’s modulus, the stress and strain at yield, and the stress and strain at break were evaluated at a strain rate of 1 min^−1^ in an ambient environment of 22 ± 1 °C and 35 ± 7% RH. The average values were based on five independent measurements.

## 3. Results and Discussion

### 3.1. Feedstock Characterization

**Figure 2:**
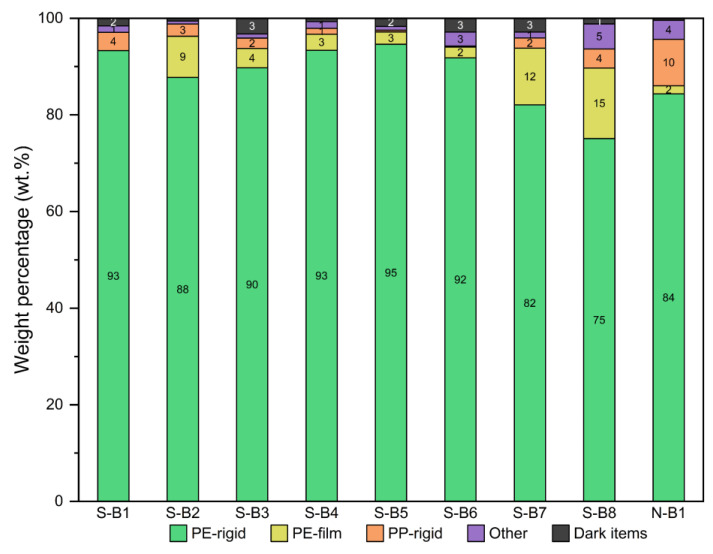
Feedstock distribution of identified components, polymeric content, and dark items given as mass percentages in each sample batch.

The polymer composition of the rigid PE fraction sorted from the plastics packaging waste was assessed, and the results are shown in Figure 2. All the studied PE batches were contaminated with PP, often originating from a PP cap on a PE bottle.

Based on the eight sampled batches of the sorted PE-rigid stream from plant S, the average PE content (PE-rigid and PE-film) was 94 ± 2 wt.% at the 95% confidence level during the sampling period. The rest was 2 ± 1.2 wt.% PP, of which 1.6 ± 1.1 wt.% was PP-caps, 1.8 ± 1.3 wt.% other polymers or non-polymers, and 1.8 ± 0.9 wt.% items that could not be identified with NIR due to their dark color. The sampled batch of the sorted PE-rigid stream from plant N had an average PE content of 86 wt.%, the rest being 9.6 wt.% PP, of which 7.9 wt.% was PP-caps, 3.9 wt.% other, and 0.4 wt.% dark items. Brouwer et al. [49] reported similar distributions of polymeric and non-polymeric materials in a sample of post-consumer rigid PE waste in The Netherlands, and they pointed out that the polymeric contamination was mainly design related because two or more polymers were used in a single product.

### 3.2. Sample Characterization

#### 3.2.1. Thermal Properties

There were only minor differences between the pellets and the injection-molded samples with regard to the thermal properties; thus, only the results of the pellets are reported here. The first heating endotherms were all rather similar with a main peak at 132–135 °C, related to high-density polyethylene (HDPE), and a small peak between 161 and 163 °C attributed to the polypropylene (PP) fraction [8,50]. As with the feedstock characterization, the samples from plant N exhibited a slightly higher PP peak indicating the larger amount of PP in the material. Neither the washing medium nor the compounding temperature had any significant influence on the melting characteristics. The estimated crystallinity of the HDPE fraction, based on the feedstock characterization, was similar for all the samples: 64–67% for the laboratory-scale washed samples from plant S (S_LW), 69–71% for the laboratory-scale washed samples from plant N (N_LW), and 67–69% for the large-scale high-intensity washed samples from plant N (N_IW). These values were similar to those reported for recycled HDPE samples in several studies [8,15,42].

The average ash content was 2.2 ± 0.4 wt.% for all the samples and probably originated from fillers such as calcium carbonate, pigments such as titanium dioxide, and traces of catalysts from the polymer synthesis [8,15,51,52,53].

The oxidation temperatures (*T_ox_*), given in Table 2, were similar for the laboratory-scale washed samples from plants S and N, whereas the large-scale high-intensity washed samples from plant N showed slightly higher values. The higher *T_ox_* of the latter samples could be due to the removal of low-stabilized PE-film during large-scale washing. The *T_ox_* decreased with increasing compounding temperature in all the sample groups. Detergent washing produced higher *T_ox_* values in both the laboratory-scale washed samples. The reason for this is unclear, but it may be due to different losses of thermo-oxidative stabilizers during washing. This, however, would require further studies. All the samples had a *T_ox_* between 202 and 229 °C, which indicated some remaining stabilizer activity, considering that the unstabilized virgin PE had a *T_ox_* of 180 ± 5 °C [54]. The results also indicated that the activity of the residual stabilizers decreased with increasing compounding temperature.

#### 3.2.2. Molecular Structure

The weight average molecular mass (*M_w_*) and the polydispersity (*PD*) were in similar ranges for each group of samples. The laboratory-scale water-washed samples from plant S showed a decrease in both *M_w_* and *PD* with increasing compounding temperature, which suggested that chain scission occurred [9,12,20]. Washing with a detergent resulted in the lowest *M_w_* but a slightly higher *PD*, which supports the hypothesis of Santana and Gondim [10] that the detergent-related degradation occurred mainly as chain scission and led to a broadening of the molecular weight distribution. This pattern was not, however, observed in the laboratory-scale washed samples from plant N. Both water- and detergent-washed samples compounded at 240 °C exhibited the highest *M_w_*, and the latter had a slightly higher *PD.* The *M_w_* was lower for the laboratory-scale water-washed sample from plant N compounded at 200 °C, which was further reduced at 260 °C. The *M_w_* of the large-scale high-intensity washed samples from plant N resulted in a similar range (±10 kDa) as the laboratory-scale washed samples from plant N and exhibited a similar pattern, i.e., the sample had the highest *M_w_* when compounded at 240 °C and the lowest when compounded at 260 °C. This pattern may be a result of the changing balance between chain scission and recombination of reactive chain ends at molecular degradation. However, the reason for this behavior is still unclear and needs further investigation.

### 3.3. Rheological Properties

**Figure 3:**
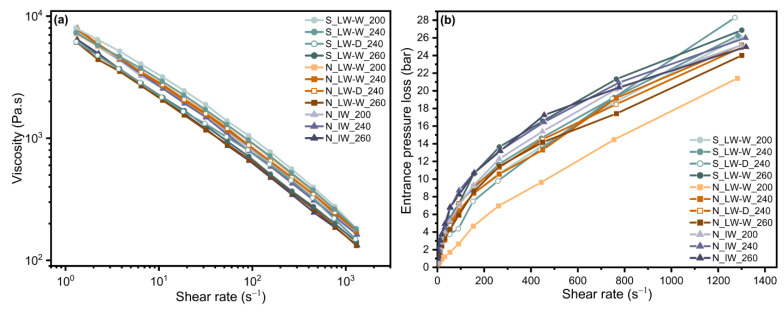
(**a**) The viscosity versus the shear rate at 240 °C for all the samples. (**b**) The entrance pressure loss versus the shear rate at 240 °C for all the samples.

The melt-mass-flow rate (*MFR*) for all the samples varied in a narrow range from 0.4 to 1.3 g/10 min as seen in previous studies [55,56], and the melt viscosity was therefore also plotted versus the shear rate, as shown in Figure 3a. The entrance pressure losses in the capillary are also given as a function of the shear rate in Figure 3b.

The viscosity levels in the shear rate region agreed in general with the molecular mass results, i.e., the viscosity decreased with decreasing *M_w_* value in each group of samples. The samples compounded with the highest temperature profile clustered at the lower end of the viscosity range. These observations strengthen the suggestion that the degradation increased with a higher compounding temperature, as was also reflected in lower *T_ox_* values.

The pressure losses in capillary flow, shown in Figure 3b, clearly indicate that the laboratory-scale water-washed sample from plant N compounded at 200 °C exhibited the lowest pressure loss, indicating a significantly lower melt elasticity [57]. The other samples were in a similar range, the difference in general being ±4 bar. The large-scale high-intensity washed samples from plant N exhibited, however, slightly greater entrance pressure losses than the laboratory-scale washed samples from plant N.

**Figure 4:**
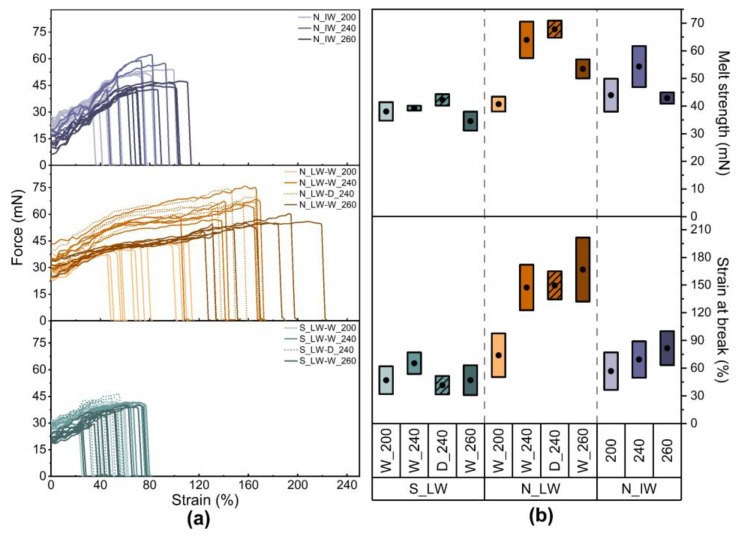
(**a**) Force–strain curves for some of the melts at 240 °C. (**b**) Melt strength and strain at break of the melts, where a dot in a box shows the mean, and the length of the box shows the standard deviation.

The force–strain plots of the melts are shown in Figure 4a, and the melt strength and strain at break of the melts are given in Figure 4b. The laboratory-scale washed samples from plants S and N showed a clear difference in melt extensibility. In general, the laboratory-scale washed samples from plant N had a higher strain at break than those from plant S. This observation agreed with the entrance pressure losses. In general, the laboratory-scale washed samples from plant N showed lower pressure losses, which implied a greater drawability.

Neither the washing medium nor the compounding temperature had any great influence on the samples from plant S with regard to either the strain at break or the melt strength, the latter being of the order 40 mN. The greater extensibility of the laboratory-washed samples from plant N has already been pointed out. It also increased with increasing compounding temperature, whereas the washing medium appeared to have no major effect. Compared with the plant S material, the melt strength was significantly higher for the samples from plant N when the compounding temperature was set to 240 °C, which parallels the *M_w_* values given in Table 2.

The strain at break of the large-scale high-intensity washed samples from plant N was of the same magnitude as that of the samples from plant S, and there was no clear effect of the compounding temperature, possibly because sufficient thermo-oxidative stabilizer activity remained. These samples also exhibited a somewhat higher melt strength than those of the other samples, the highest value being obtained with a compounding temperature of 240 °C, which again corresponded to a higher *M_w_* (Table 2). The differences in melt behavior between laboratory-scale and large-scale high-intensity washing may be due to a change in polymer properties and structure after the NaOH treatment, but this would need a more detailed study.

**Figure 5:**
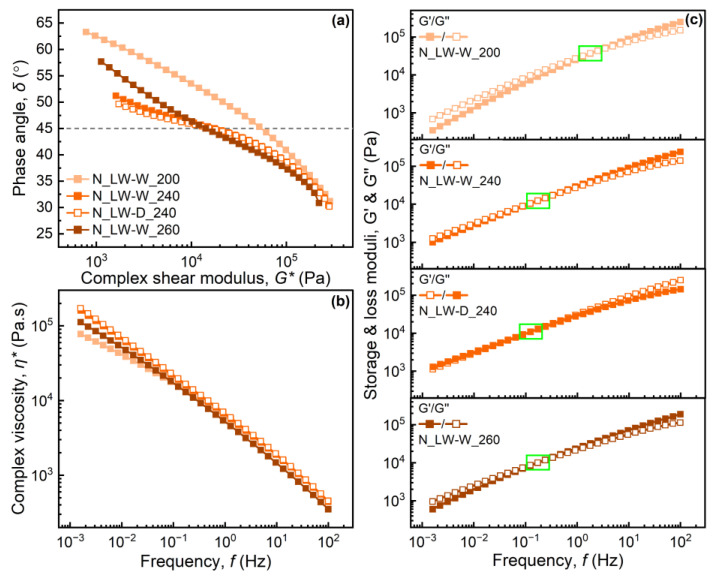
Dynamic rheological behavior of N_LW samples in frequency sweeps: (**a**) a van Gurp–Palmen plot showing the phase angle as a function of the complex modulus. (**b**) Complex viscosity as a function of frequency. (**c**) Storage and loss moduli as functions of frequency for each sample with a crossover point indicated by a green rectangle.

Figure 5 shows the results of the oscillatory shear measurements: (a) phase angle vs. complex shear modulus, known as a Van Gurp–Palmen plot; (b) complex viscosity vs. frequency; and (c) storage and loss moduli vs. frequency for the laboratory-washed samples from plant N.

The vGP plots in Figure 5a indicate that the compounding at different temperatures leads to structural differences and different levels of branching in the laboratory-scale washed samples from plant N [20,35,39]. The sample compounded with the low-temperature profile showed more linear-polymer-like behavior, i.e., a monotonic decrease, whereas both medium- and high-temperature compounding led to a curve that exhibited a shape more like that of a branched polymer, i.e., a slight bump or plateau. The shift in the phase angle to lower values in the vGP plot supports this hypothesis as it has previously been reported that it is partly due to an increase in long branches or in the molecular weight distribution [20,35]. In this case the polydispersity values did not follow the change in the phase angle, so that different levels of branching might be the greater reason for the observed differences. Water and detergent washing resulted in similar characteristics. The sample compounded at a lower temperature was more viscous than the others, all of which showed a pronounced elastic behavior. The pattern of elastic behavior in the vGP plots for these samples agreed in a sense with the entrance pressure losses in Figure 3b, where low-temperature compounding resulted in the lowest entrance pressure losses, i.e., the melt with the lowest elasticity. The crossover points, in Figure 5c, at which the storage and the loss modulus intersect, also supported this as a lower frequency value of the crossover point indicates a more elastic behavior [37]. The complex viscosity and storage modulus curves in Figure 5b,c support the vGP plot as higher values of both complex viscosity and storage modulus at lower frequencies indicate a more branched structure, and it can therefore be concluded that the samples contained different levels of branching, although a quantitative study of the long- or short-chain branching requires a more detailed analysis.

### 3.4. Mechanical Properties

**Figure 6:**
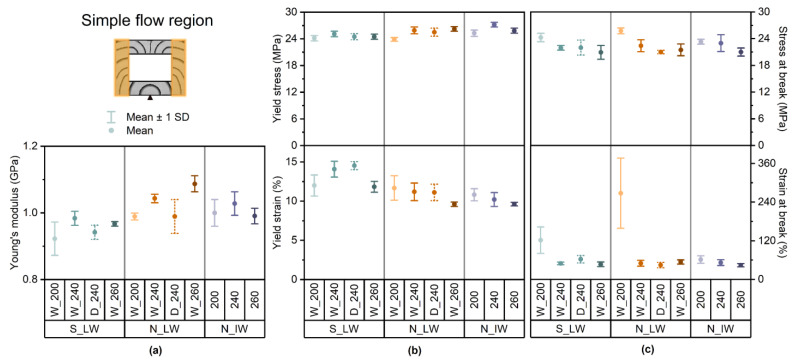
Mechanical properties of the samples in the simple flow region of the IM frame: (**a**) Young’s modulus, (**b**) yield stress and strain, and (**c**) stress and strain at break.

Figure 6 shows the mechanical properties (Young’s modulus, yield stress, yield strain, tensile strength at break, and elongation at break) of the samples in the simple flow region of the injection molded frame.

In the simple flow region, Young’s modulus (Figure 6a) differed only slightly between the different sources and within each of these groups. In general, the samples from plant N were slightly more stiff than the samples from plant S. Although the differences were minor, the Young’s modulus increased with increasing compounding temperature for the laboratory-scale washed samples from plant N. For the laboratory-scale washed samples from plant S and the large-scale high-intensity washed samples from plant N, the compounding at 240 °C resulted in the highest stiffness. For both the plant S and N samples, the detergent washing resulted in a slightly lower Young’s modulus. The yield stresses and strains (Figure 6b) were 24–27 MPa and 10–15% for all the samples. Neither the compounding temperature nor the washing medium had any great influence on the yield. The stresses at break were in a similar range for all the samples, varying between 21 and 26 MPa (Figure 6c). In each group, there was a weak tendency for the tensile strength to decrease with increasing compounding temperature. The detergent washing had no influence on the sample from plant S and only slightly reduced the tensile strength of the sample from plant N. The low-temperature compounded laboratory-scale washed samples from both plants S and N had the highest average strain at break, 122% and 268%, respectively. A higher compounding temperature and detergent washing resulted in a significant reduction in the ultimate strain, but there was no significant difference between the groups.

Similar patterns were observed in both the weld line and gate regions as in the simple flow region. Overall, the weld line region exhibited the lowest mechanical properties, especially in the stress- and strain-at-break values. The yield stresses and strains were lower in the gate region than in the simple flow region. In contrast, the stresses and strains at break were higher in the gate region for all the samples. Both the stiffness and strength values of the recycled materials studied in this work were, however, in the range of the values for virgin polymers reported for applications such as bottle and rigid injection molding, although the elongation at break was lower than the normal values (200–300%) for virgin polymers, especially when the material were compounded at higher temperatures [58]. Here, it can be commented that further use of the recycled materials would likely favor extrusion processing and blow molding motivated by the expected increase in elasticity in the melt state provided by the indicated increase in chain branching.

## 4. Conclusions

Feedstock characterization indicated that most of the rigid PP contamination in the rigid PE stream originated from PP caps, which meant that the problem was related to the design of the product. Although they originated from different sources, separated household plastic waste and mixed municipal solid waste, the recycled materials exhibited similar properties with only slight differences. The compounding temperature had the strongest influence on the properties, where an increase in the compounding temperature profile led to an increased degradation, reflected in the oxidation temperature, molecular mass, and viscosity. It was also evident that structural changes such as in, e.g., the different level of branching occurred when the samples were compounded at different temperatures. The solid-state stress- and strain-at-break values decreased with increasing compounding temperature in all cases, but no significant influence of the processing temperature was observed on the thermal properties, melt elasticity, Young’s modulus, and yield stress and strain of the samples. Detergent washing had no pronounced effect on the properties of the studied material, apart from a maintained oxidation temperature. Large-scale high-intensity washing produced properties similar to those of laboratory-scale washing but resulted in a higher oxidation temperature, higher entrance pressure losses, and a lower strain at break in both the molten and solid states, which indicated some structural differences probably due to the different feedstock composition either from the beginning or as a result of the different washing treatment.

## Figures and Tables

**Table 1:** The notations and processing histories of the injection-molded samples.

Material Origin	Washing	Compounding T Profile (°C)	Sample Code
Source-separated household plastic packaging waste in Sweden, plant S	Laboratory-scale washing with water only	110-160-200-200-200-210	S_LW-W_200
	Laboratory-scale washing with water only	110-160-200-240-240-250	S_LW-W_240
	Laboratory-scale washing with detergent	110-160-200-240-240-250	S_LW-D_240
	Laboratory-scale washing with water only	110-160-210-260-260-270	S_LW-W_260
Mixed municipal solid waste in Norway, plant N	Laboratory-scale washing with water only	110-160-200-200-200-210	N_LW-W_200
	Laboratory-scale washing with water only	110-160-200-240-240-250	N_LW-W_240
	Laboratory-scale washing with detergent	110-160-200-240-240-250	N_LW-D_240
	Laboratory-scale washing with water only	110-160-210-260-260-270	N_LW-W_260
	Large-scale high-intensity washing	110-160-200-200-200-210	N_IW_200
	Large-scale high-intensity washing	110-160-200-240-240-250	N_IW_240
	Large-scale high-intensity washing	110-160-210-260-260-270	N_IW_260

**Table 2:** Thermal, structural, and rheological properties of the samples.

Sample	*T_ox_* (°C)	*M_w_* (kDa)	*PD*	*MFR* (g/10 min)
S_LW-W_200	215	133	6.5	0.6
S_LW-W_240	209	121	6.1	0.7
S_LW-D_240	222	75	7.0	0.7
S_LW-W_260	202	92	5.8	1.1
N_LW-W_200	215	93	6.8	1.0
N_LW-W_240	213	132	7.1	0.5
N_LW-D_240	218	132	8.6	0.6
N_LW-W_260	202	74	5.6	1.3
N_IW_200	229	106	6.7	0.7
N_IW_240	226	123	6.9	0.4
N_IW_260	218	84	7.0	0.6

## Data Availability

Data are contained within the article.

## References

[1] European Parliament and the Council Directive (EU) 2018/852 of the European Parliament and of the Council of 30 May 2018 Amending Directive 94/62/EC on Packaging and Packaging Waste. https://eur-lex.europa.eu/legal-content/EN/TXT/?uri=CELEX%3A32018L0852.

[2] Schyns Z.O.G., Shaver M.P. (2020). Mechanical Recycling of Packaging Plastics: A Review. Macromol. Rapid Commun..

[3] Shen L., Worrell E., Worrell E., Reuter M.A. (2014). Plastic Recycling. Handbook of Recycling: State-of-the-art for Practitioners, Analysts, and Scientists.

[4] Plastics Europe EPRO Plastics—The Facts 2022. https://plasticseurope.org/knowledge-hub/plastics-the-facts-2022/.

[5] Faraca G., Astrup T. (2019). Plastic Waste from Recycling Centres: Characterisation and Evaluation of Plastic Recyclability. Waste Manag..

[6] Roosen M., Mys N., Kusenberg M., Billen P., Dumoulin A., Dewulf J., Van Geem K.M., Ragaert K., De Meester S. (2020). Detailed Analysis of the Composition of Selected Plastic Packaging Waste Products and Its Implications for Mechanical and Thermochemical Recycling. Environ. Sci. Technol..

[7] Thoden van Velzen E.U., Chu S., Alvarado Chacon F., Brouwer M.T., Molenveld K. (2020). The Impact of Impurities on the Mechanical Properties of Recycled Polyethylene. Packag. Technol. Sci..

[8] Alvarado Chacon F., Brouwer M.T., Thoden van Velzen E.U., Smeding I.W. (2020). A First Assessment of the Impact of Impurities in PP and PE Recycled Plastics.

[9] Rosli N.A., Ahmad I., Parameswaranpillai J., Rangappa S.M., Rajkumar A.G., Siengchin S. (2021). Mechanical Properties of Recycled Plastics. Recent Developments in Plastic Recycling. Composites Science and Technology.

[10] Santana R.M.C., Gondim G. (2009). Influence of Cleaning Conditions on the Degradation of Recycled HDPE. J. Appl. Polym. Sci..

[11] Gijsman P. (2008). Review on the Thermo-Oxidative Degradation of Polymers during Processing and in Service. e-Polymers.

[12] Vilaplana F., Karlsson S. (2008). Quality Concepts for the Improved Use of Recycled Polymeric Materials: A Review. Macromol. Mater. Eng..

[13] Oblak P., Gonzalez-Gutierrez J., Zupančič B., Aulova A., Emri I. (2016). Mechanical Properties of Extensively Recycled High-Density Polyethylene. Conference Proceedings of the Society for Experimental Mechanics Series.

[14] Rudolph N., Kiesel R., Aumnate C. (2020). Kunststoffrecycling—Schonung Wertvoller Ressourcen. Einführung Kunststoffrecycling; Ökonomische, ökologische und technische Aspekte der Kunststoffabfallverwertung.

[15] Alzerreca M., Paris M., Boyron O., Orditz D., Louarn G., Correc O. (2015). Mechanical Properties and Molecular Structures of Virgin and Recycled HDPE Polymers Used in Gravity Sewer Systems. Polym. Test..

[16] Andersson T., Stålbom B., Wesslén B. (2003). Degradation of Polyethylene During Extrusion. II. Degradation of Low-Density Polyethylene, Linear Low-Density Polyethylene, and High-Density Polyethylene in Film Extrusion. J. Appl. Polym. Sci..

[17] Cecon V.S., Da Silva P.F., Vorst K.L., Curtzwiler G.W. (2021). The Effect of Post-Consumer Recycled Polyethylene (PCRPE) on the Properties of Polyethylene Blends of Different Densities. Polym. Degrad. Stab..

[18] Choudhury A., Mukherjee M., Adhikari B. (2005). Thermal Stability and Degradation of the Post-Use Reclaim Milk Pouches during Multiple Extrusion Cycles. Thermochim. Acta.

[19] Dahlbo H., Poliakova V., Mylläri V., Sahimaa O., Anderson R. (2018). Recycling Potential of Post-Consumer Plastic Packaging Waste in Finland. Waste Manag..

[20] Dordinejad A.K., Sharif F., Ebrahimi M., Rashedi R. (2018). Rheological and Thermorheological Assessment of Polyethylene in Multiple Extrusion Process. Thermochim. Acta.

[21] Dostál J., Kašpárková V., Zatloukal M., Muras J., Šimek L. (2008). Influence of the Repeated Extrusion on the Degradation of Polyethylene. Structural Changes in Low Density Polyethylene. Eur. Polym. J..

[22] Jin H., Gonzalez-Gutierrez J., Oblak P., Zupančič B., Emri I. (2012). The Effect of Extensive Mechanical Recycling on the Properties of Low Density Polyethylene. Polym. Degrad. Stab..

[23] Moss S., Zweifel H. (1989). Degradation and Stabilization of High Density Polyethylene during Multiple Extrusions. Polym. Degrad. Stab..

[24] Oblak P., Gonzalez-Gutierrez J., Zupančič B., Aulova A., Emri I. (2015). Processability and Mechanical Properties of Extensively Recycled High Density Polyethylene. Polym. Degrad. Stab..

[25] Pinheiro L.A., Chinelatto M.A., Canevarolo S.V. (2006). Evaluation of Philips and Ziegler-Natta High-Density Polyethylene Degradation during Processing in an Internal Mixer Using the Chain Scission and Branching Distribution Function Analysis. Polym. Degrad. Stab..

[26] Bagley E.B., Schreiber H.P., Eirich F.R. (1969). Elasticity Effects in Polymer Extrusion. Rheology.

[27] Micic P., Bhattacharya S.N. (2000). Rheology of LLDPE, LDPE and LLDPE/LDPE Blends and Its Relevance to the Film Blowing Process. Polym. Int..

[28] Münstedt H. (2019). Elastic Behavior and Processing of Polymer Melts. AIP Conf. Proc..

[29] Plochocki A.P., Czarnecki L. (1990). Implications of The Melt Elasticity of LDPE in The Film Blowing Process. J. Plast. Film Sheeting.

[30] Polychronopoulos N.D., Vlachopoulos J., Jafar Mazumder M., Sheardown H., Al-Ahmed A. (2019). Polymer Processing and Rheology. Functional Polymers. Polymers and Polymeric Composites: A Reference Series.

[31] Wagner M.H., Schulze V., Göttfert A. (1996). Rheotens-Mastercurves and Drawability of Polymer Melts. Polym. Eng. Sci..

[32] La Mantia F.P., Acierno D. (1985). Influence of the Molecular Structure on the Melt Strength and Extensibility of Polyethylenes. Polym. Eng. Sci..

[33] Van Gurp M., Palmen J. (1998). Time-Temperature Superposition for Polymeric Blends. Rheol. Bull..

[34] Trinkle S., Friedrich C. (2001). Van Gurp-Palmen-Plot A Way to Characterize Polydispersity of Linear Polymers. Rheol. Acta.

[35] Agrawal P., Silva M.H.A., Cavalcanti S.N., Freitas D.M.G., Araújo J.P., Oliveira A.D.B., Mélo T.J.A. (2022). Rheological Properties of High-Density Polyethylene/Linear Low-Density Polyethylene and High-Density Polyethylene/Low-Density Polyethylene Blends. Polym. Bull..

[36] Barroso V.C., Maia J.M. (2005). Influence of Long-Chain Branching on the Rheological Behavior of Polyethylene in Shear and Extensional Flow. Polym. Eng. Sci..

[37] Shahi P., Behravesh A.H., Haghtalab A., Rizvi G., Pop-Iliev R., Goharpei F. (2016). Effect of Mixing Intensity on Foaming Behavior of LLDPE/HDPE Blends in Thermal Induced Batch Process. Polym. Plast. Technol. Eng..

[38] Wood-Adams P.M., Dealy J.M., DeGroot A.W., Redwine O.D. (2000). Effect of Molecular Structure on the Linear Viscoelastic Behavior of Polyethylene. Macromolecules.

[39] Trinkle S., Walter P., Friedrich C. (2002). Van Gurp-Palmen Plot II—Classification of Long Chain Branched Polymers by Their Topology. Rheol. Acta.

[40] (2016). Plastics—Differential Scanning Calorimetry (DSC)—Part 1: General Principles.

[41] (2018). Plastics—Differential Scanning Calorimetry (DSC)—Part 6: Determination of Oxidation Induction Time (Isothermal OIT) and Oxidation Induction Temperature (Dynamic OIT).

[42] Mylläri V., Hartikainen S., Poliakova V., Anderson R., Jönkkäri I., Pasanen P., Andersson M., Vuorinen J. (2016). Detergent Impurity Effect on Recycled HDPE: Properties after Repetitive Processing. J. Appl. Polym. Sci..

[43] (2022). Plastics—Determination of the Melt Mass-Flow Rate (MFR) and Melt Volume-Flow Rate (MVR) of Thermoplastics—Part 1: Standard Method.

[44] (2021). Plastics—Determination of the Fluidity of Plastics Using Capillary and Slit-Die Rheometers.

[45] (2021). Plastics—Determination of the Drawing Characteristics of Thermoplastics in the Molten State.

[46] Thunwall M., Boldizar A., Rigdahl M., Kuthanová V. (2006). On the Stress-Strain Behavior of Thermoplastic Starch Melts. Int. J. Polym. Anal. Charact..

[47] (2012). Plastics—Determination of Tensile Properties—Part 2: Test Conditions for Moulding and Extrusion Plastics.

[48] (2012). Plastics—Determination of Tensile Properties—Part 1: General Principles.

[49] Brouwer M.T., Thoden van Velzen E.U., Augustinus A., Soethoudt H., De Meester S., Ragaert K. (2018). Predictive Model for the Dutch Post-Consumer Plastic Packaging Recycling System and Implications for the Circular Economy. Waste Manag..

[50] Lechner M.D., Warlimont H., Martienssen W. (2018). Polymers. Springer Handbook of Materials Data. Springer Handbooks.

[51] Cuadri A.A., Martín-Alfonso J.E. (2017). The Effect of Thermal and Thermo-Oxidative Degradation Conditions on Rheological, Chemical and Thermal Properties of HDPE. Polym. Degrad. Stab..

[52] Gall M., Wiener M., Chagas de Oliveira C., Lang R.W., Hansen E.G. (2020). Building a Circular Plastics Economy with Informal Waste Pickers: Recyclate Quality, Business Model, and Societal Impacts. Resour. Conserv. Recycl..

[53] Möllnitz S., Feuchter M., Duretek I., Schmidt G., Pomberger R., Sarc R. (2021). Processability of Different Polymer Fractions Recovered from Mixed Wastes and Determination of Material Properties for Recycling. Polymers.

[54] Karlsson K., Assargren C., Gedde U.W. (1990). Thermal Analysis for the Assessment of Antioxidant Content in Polyethylene. Polym. Test..

[55] Kealy T. (2009). Rheological Analysis of the Degradation of HDPE during Consecutive Processing Steps and for Different Processing Conditions. J. Appl. Polym. Sci..

[56] Shenoy A.V., Chattopadhyay S., Nadkarni V.M. (1983). From Melt Flow Index to Rheogram. Rheol. Acta.

[57] La Nieve H.L., Bogue D.C. (1968). Correlation of Capillary Entrance Pressure Drops with Normal Stress Data. J. Appl. Polym. Sci..

[58] Demets R., Van Kets K., Huysveld S., Dewulf J., De Meester S., Ragaert K. (2021). Addressing the Complex Challenge of Understanding and Quantifying Substitutability for Recycled Plastics. Resour. Conserv. Recycl..

